# Causal effects of non-alcoholic fatty liver disease on osteoporosis: a Mendelian randomization study

**DOI:** 10.3389/fendo.2023.1283739

**Published:** 2023-12-12

**Authors:** Yue Zhou, Yunzhi Ni, Zhihong Wang, Gerald J. Prud’homme, Qinghua Wang

**Affiliations:** ^1^ Department of Endocrinology and Metabolism, Huashan Hospital, Shanghai Medical School, Fudan University, Shanghai, China; ^2^ Department of Laboratory Medicine and Pathobiology, University of Toronto, Toronto, ON, Canada

**Keywords:** Mendelian randomization, causality, non-alcoholic fatty liver disease, liver fat content, osteoporosis

## Abstract

**Background:**

Osteoporosis (OP) is a systemic skeletal disease characterized by compromised bone strength leading to an increased risk of fracture. There is an ongoing debate on whether non-alcoholic fatty liver disease (NAFLD) is an active contributor or an innocent bystander in the pathogenesis of OP. The aim of this study was to assess the causal association between NAFLD and OP.

**Methods:**

We performed two‐sample Mendelian randomization (MR) analyses to investigate the causal association between genetically predicted NAFLD [i.e., imaging‐based liver fat content (LFC), chronically elevated serum alanine aminotransferase (cALT) and biopsy-confirmed NAFLD] and risk of OP. The inverse variant weighted method was performed as main analysis to obtain the causal estimates.

**Results:**

Imaging-based LFC and biopsy-confirmed NAFLD demonstrated a suggestive causal association with OP ([odds ratio (OR): 1.003, 95% CI: 1.001-1.004, P < 0.001; OR: 1.001, 95% CI: 1.000-1.002, P = 0.031]). The association between cALT and OP showed a similar direction, but was not statistically significant (OR: 1.001, 95% CI: 1.000-1.002, P = 0.079). Repeated analyses after exclusion of genes associated with confounding factors showed consistent results. Sensitivity analysis indicated low heterogeneity, high reliability and low pleiotropy of the causal estimates.

**Conclusion:**

The two‐sample MR analyses suggest a causal association between genetically predicted NAFLD and OP.

## Introduction

Osteoporosis (OP) is a slowly progressing systemic metabolic bone disease caused by the imbalance between bone formation and bone resorption (1). The typical features of OP include bone loss, bone microstructure destruction and increased bone fragility, rendering patients prone to low-energy fractures (1). The global prevalence of OP was estimated to be 19.7%, which varied according to countries (from 4.1% in Netherlands to 52.0% in Turkey) and continents (from Oceania 8.0% to 26.9% in Africa) (2). In the United States, approximately 53.6 million Americans suffer from OP and approximately 2 million osteoporotic fractures occur annually (3), with related costs exceeding 95 billion dollars per year (4, 5). This condition greatly increases the economic costs and has become a major public health problem, highlighting the importance of taking measures to reduce the risk of OP. Therefore, identifying independent risk factors for OP and osteoporotic fractures, as well as patients who should be targeted with more intensive therapy has important implications.

Numerous risk factors for OP have been identified, including increasing age, female, sex hormone deficiency, body size, smoking, low physical activity, low vitamin D intake, use of certain drugs (e.g. glucocorticoids) and some chronic medical conditions (e.g. hypertension, diabetes) (6). Recent clinical studies revealed that non-alcoholic fatty liver disease (NAFLD) is associated with an increased risk of OP and osteoporotic fractures (7, 8). NAFLD is a spectrum of chronic liver diseases including fatty liver, bland steatosis, lobular necro-inflammation, and more aggressive non-alcoholic steatohepatitis (NASH), fibrosis and cirrhosis (9, 10). With a global prevalence of 25.2%, NAFLD has come to the forefront as one of the major causes of chronic liver disease (11), which not only increased the risk for end-stage liver disease and hepatocellular carcinoma, but also raised the danger of incident diabetes (12) and secondary cardiovascular diseases (13). However, some observational studies have reported no association between OP and NAFLD (14, 15). Therefore, conflicting evidence regarding the association between NAFLD and OP has been obtained thus far.

Despite the evidence on the association between NAFLD and OP, there is an ongoing discussion on whether NAFLD actively contributes to OP or is just an innocent bystander. It should be mentioned that NAFLD is related to risk factors of OP, such as type 2 diabetes (T2D) (12), which could be confounders in the assessment of their causal relationship. Mendelian randomization (MR) analysis, which uses genetic variants as instrumental variables (IVs), is a powerful statistical tool for investigating causal relationships which have grown in popularity in epidemiology (16). Genetic variants, which are unrelated to environmental factors, are randomly distributed at conception, which minimizes confounding and reverse causality. Here, we used two-sample MR analyses to assess the potential causal relationship between NAFLD and the risk of OP.

## Methods

We performed two‐sample MR analyses with available large-scale summary‐level data, which were derived from publicly available genome‐wide association study (GWAS) in which ethical approval and informed consent were provided. Therefore, no separate ethical approval was required for this study.

### Study design

**Figure 1:**
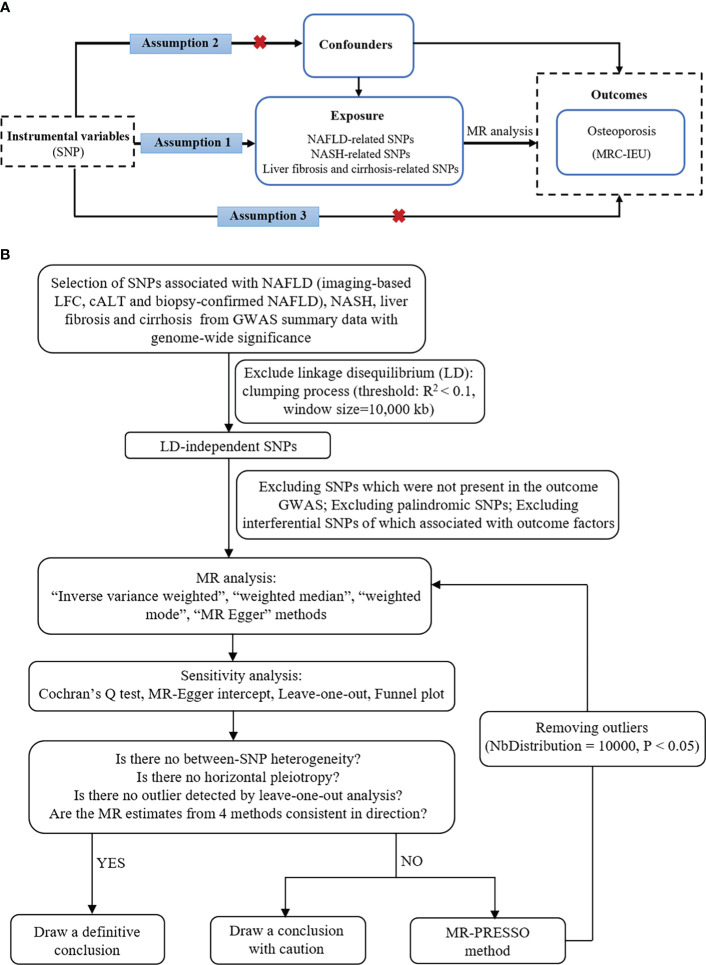
The basic principles of MR study. **(A)** Three principal assumptions. **(B)** Study flow diagram. To demonstrate causal estimates from genetically predicted exposures, MR analysis requires to meet three key assumptions. The relevance assumption is that the genetic instrument should be strongly related to the exposure of interest, which was attained as the study instrumented genome-wide significant SNPs from previous GWAS. The independence assumption is that the genetic effect should not be driven by a confounder. The exclusivity assumption means that there is no direct effect from a SNP to outcome. cALT, chronically elevated serum alanine aminotransferase; GWAS, genome-wide association studies; LFC, liver fat content; MR, Mendelian randomization; NAFLD, non-alcoholic fatty liver disease; NASH, non-alcoholic steatohepatitis; SNP, single-nucleotide polymorphism.

In this study, NAFLD was used as the exposure factor, and single nucleotide polymorphisms (SNPs) significantly related to NAFLD were used as IVs. OP was used as the outcome variable. Assumption 1 is that there is a significant correlation between the IV and the exposure factor; Assumption 2 is that the IV was not associated with any exposure-outcome confounding factor; Assumption 3 is that the IV does not affect the outcome unless it is possible to do so through association with exposure (**Figure 1A**).

### Selection of instrumental variables

We used genetic variants associated with three different NAFLD-related exposures, including imaging-based liver fat content (LFC), chronically elevated serum alanine aminotransferase (Calt), and biopsy‐confirmed NAFLD. We obtained gene‐exposure data from the UK Biobank for the imaging study of LFC (GWAS ID: GCST90016673), in which we included 32,859 participants of European ancestry who underwent the MRI scan (17). For each instrument, we chose independent, genome‐wide significant (P < 5×10^-8^) variants associated with LFC, which included 26 SNPs after removing linkage disequilibrium. We also used data derived from a recently published GWAS for Calt (GWAS ID: GCST90129601), in which NAFLD was defined as an elevated ALT > 40 U/L for men or >30 U/L for women during at least two time points at least 6 months apart within a 2‐year period, after exclusion of other liver diseases (18). This study reported 77 independent, genome‐wide significant SNPs in the discovery cohort (the Million Veteran Program), which included 90,408 cases and 128,187 controls of four ancestral groups (European‐Americans, African‐Americans, Hispanic‐Americans, and Asian‐Americans). Of these, 36 SNPs were subsequently replicated in another external validation cohort which is biopsy‐confirmed NAFLD (7,397 cases and 56,785 controls). Considering that NASH, fibrosis and cirrhosis are clinically important phenotypes in NAFLD and are more closely related to clinical complications, we furthermore collected data of NASH (157 cases and 377,120 controls) and liver fibrosis and cirrhosis (1,841 cases and 366,450 controls) from Ristey FinnGen website (https://r9.risteys.finngen.fi/endpoints/NASH, https://r9.risteys.finngen.fi/endpoints/K11_FIBROCHIRLIV). In the FinnGen study, NAFLD-related diseases were defined using the International Classification of Disease (ICD) code.

In this MR study, the following sets of IVs were used: (i) Imaging-based LFC‐associated SNPs (n = 26); (ii) Calt‐associated SNPs (n = 77); (iii) Calt‐associated SNPs with nominal significance and directional concordance in the biopsy cohorts (n = 36; the effect estimates for the biopsy data were used); (iv) NASH (n = 1); (v) Liver fibrosis and cirrhosis (n = 1). We used strict selection criteria to obtain qualified IVs. First, the clump (linkage disequilibrium r^2^ < 0.1, kb = 10,000) was conducted to ensure the independence among SNPs and remove the linkage disequilibrium. Second, because the independence assumption requires independence between IVs and confounding factors, SNPs associated with potential confounders were further excluded. The potential confounders associated with the selected SNPs were analyzed using the PhenoScanner database (http://www.phenoscanner.medschl.cam.ac.uk/). Third, palindromic SNPs with intermediate allele frequencies were excluded. We also calculated the F value to ensure the strength of the IVs in MR analyses, and only SNPs with an F-statistic of more than 10 were considered reliable (19). Finally, the remaining SNPs were used for MR analysis (**Figure 1B**). These SNPs are shown in detail in **Supplementary Materials 1–3** (**Supplementary Materials 1–3** showed SNPs for the effects of imaging-based LFC, Calt, and biopsy-confirmed NAFLD on OP, respectively).

### Genetic associations with outcomes

The outcome data of OP were obtained from the main results of the public GWAS dataset (http://gwas-api.mrcieu.ac.uk/, GWAS ID: ukb-b-12141). The dataset was constructed by the MRC Integrated Epidemiology Unit consortium using Biobank UK, which included 462,933 Europeans (7,547 cases and 455,386 controls) with 9,851,867 SNPs. The detailed information of the included GWAS is presented in **Table 1**.

**Table 1:** Overview of gene-exposure and gene-outcome databases used for Mendelian randomization.

GWAS ID	GWAS dataset	Phenotype	Sample size	Ethnicity	Year
ebi-a-GCST90016673	UK Biobank	Liver fat derived from abdominal MRI using deep learning	32,858	European	2021
ebi-a-GCST90129601	The Million Veteran Program	NAFLD defined as elevated ALT > 40 U/L for men or > 30 U/L for women during at least two time points at least 6 months apart within a 2-year window period at any point prior to enrolment after the exclusion of other causes of liver disease	218,595 (90,408 cases and 128,187 controls)	European-Americans (75.1%), African-Americans (17.1%), Hispanic-Americans (6.9%) and Asian-Americans (0.9%)	2022
–	FinnGen	NASH	377,277 (157 cases and 377,120 controls)	European	2022
–	FinnGen	Liver fibrosis and cirrhosis	368,291 (1,841 cases and 366,450 controls)	European	2022
ukb-b-12141	MRC-IEU	Osteoporosis	462,933 (7,547 cases and 455,386 controls)	European	2018

ALT, alanine aminotransferase; GWAS, genome‐wide association study; NAFLD, non-alcoholic fatty liver disease; NASH, non-alcoholic steatohepatitis.

### Statistical analysis

To yield an overall estimate of the causal effect of NAFLD on the risk of OP, the IVW MR analysis was used as the primary analysis for all IV sets. This method could obtain an unbiased causal estimate if there was no horizontal pleiotropy and heterogeneity (20). Three complementary MR methods, including the weighted-median, weighted mode and MR-Egger methods, were supplemented to provide a robust estimate of the association (21). Cochran’s Q statistic was calculated to quantify heterogeneity. MR‐Egger regression method was performed to assess the potential directional pleiotropy, and a statistically significant intercept will suggest directional pleiotropy which can violate the IV assumptions (22). We also examined possible pleiotropy of selected SNPs using the outlier (MR-PRESSO) test, and abnormal SNPs were removed (NbDistribution = 10,000) in order to reduce heterogeneity in the estimate of the causal effect (23). If potentially driving SNPs were found in the “leave-one-out” sensitivity analysis, conclusions should be made with caution. All analyses were performed using the R statistical software version 4.2.3 with the Two-Sample MR packages (24).

## Results

### Association between genetically predicted NAFLD and OP

**Figure 2:**
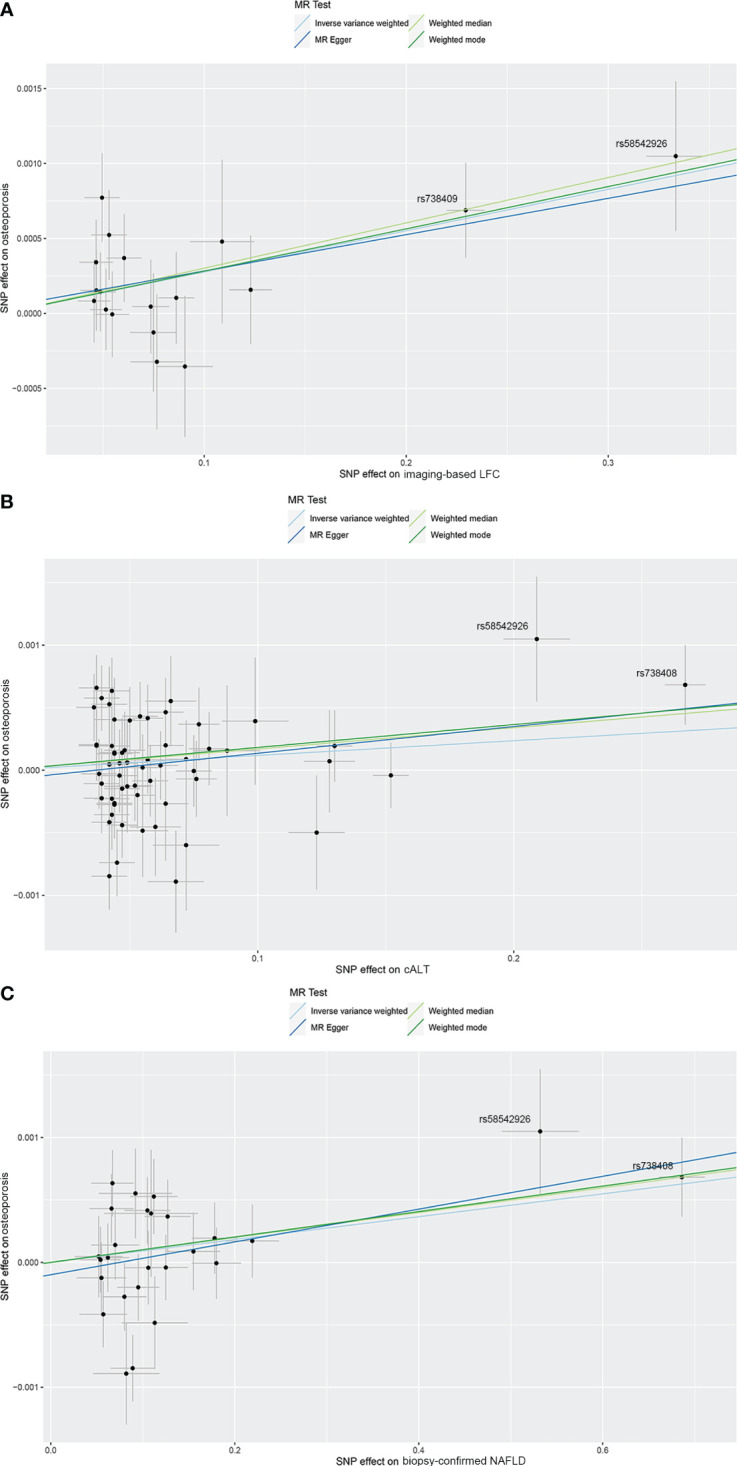
Scatterplots of the causal effect of three NAFLD-related traits on OP. **(A)** Causal effect of imaging‐based LFC on OP. **(B)** Causal effect of cALT on OP. **(C)** Causal effect of biopsy-confirmed NAFLD on OP. Analyses were conducted using IVW, weighted median, weighted mode and MR-Egger methods. The slope of each line corresponds to the estimated MR effect per method. cALT, chronically elevated serum alanine aminotransferase; IVW, inverse variance weighted; LFC, liver fat content; MR, Mendelian randomization; NAFLD, non-alcoholic fatty liver disease; OP, osteoporosis.

**Figure 3:**
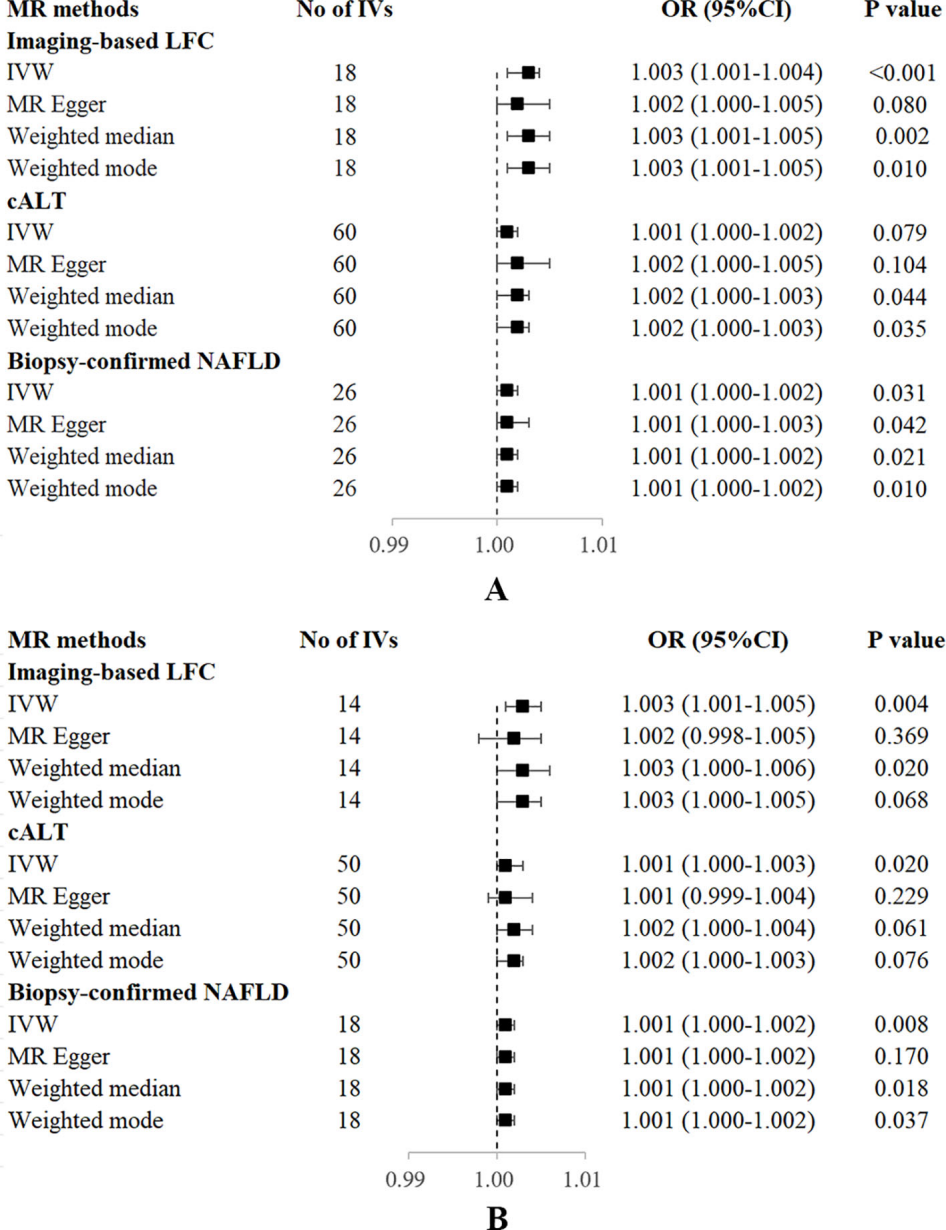
Forest plot of MR estimates of three NAFLD-related traits on OP. **(A)** without adjustment of confounding factors. **(B)** with adjustment of confounding factors. cALT, chronically elevated serum alanine aminotransferase; IVW, inverse variance weighted; LFC, liver fat content; MR, mendelian randomization; NAFLD, non-alcoholic fatty liver disease; OP, osteoporosis; OR, odds ratio; SNP, single nucleotide polymorphism.

Using 18 independent SNPs significantly associated with imaging-based LFC, the two-sample IVW MR analysis indicated a causal effect of imaging-based LFC on the risk of OP (OR: 1.003, 95% CI: 1.001-1.004, P < 0.001), which means an average 0.3% increased risk of OP per SD higher liver fat. Similar directional associations were observed with the other methods (weighted median, OR: 1.003, 95% CI: 1.001-1.005, P = 0.002; weighted mode, OR: 1.003, 95% CI: 1.001-1.005, P = 0.010), although the MR Egger analysis was not statistically significant (OR: 1.002, 95% CI: 1.000-1.005, P = 0.080; **Table 2**, **Figures 2A**, **3A**). Cochran’s Q statistic indicated low heterogeneity and high reliability (Q = 12.341, P = 0.779; **Supplementary Figure 4A**). MR-Egger regression analysis indicated no evidence of horizontal pleiotropy (intercept = 4.086e-05, P = 0.749). “Leave one-out” sensitivity analysis indicated no single SNP dominated the causal estimate in the IVW (**Supplementary Figure 5A**), and no pleiotropic outliers were detected in the MR-PRESSO analysis (P_global test_ = 0.833; **Table 3**).

**Table 2:** Causal relationship between genetically predicted NAFLD and osteoporosis.

Outcome	Exposure	IVW method		MR-Egger method		Weighted median method		Weighted mode method		IVW adjusted by confounding factors^a^	
		OR (95% CI)	P value	OR (95% CI)	P value	OR (95% CI)	P value	OR (95% CI)	P value	OR (95% CI)	P value
Osteoporosis	Imaging-based LFC	1.003 (1.001-1.004)	<0.001	1.002 (1.000-1.005)	0.080	1.003 (1.001-1.005)	0.002	1.003 (1.001-1.005)	0.010	1.003 (1.001-1.005)	0.004
	cALT	1.001 (1.000-1.002)	0.079	1.002 (1.000-1.005)	0.104	1.002 (1.000-1.003)	0.044	1.002 (1.000-1.003)	0.035	1.001 (1.000-1.003)	0.020
	Biopsy-based NAFLD	1.001 (1.000-1.002)	0.031	1.001 (1.000-1.003)	0.042	1.001 (1.000-1.002)	0.021	1.001 (1.000-1.002)	0.01	1.001 (1.000-1.002)	0.008

cALT, chronically elevated serum alanine aminotransferase; CI, confidence interval; IVW, inverse variance weighted; MR-Egger, MR-Egger regression; NAFLD, non-alcoholic fatty liver disease; OR, odds ratio.

a Confounding factors included body mass index, type 2 diabetes, smoking, and hypothyroidism.

**Table 3:** Sensitivity analyses for the causal results of the primary Mendelian randomization.

Outcome	Exposure	MR methods	No of SNPs	Cochran Q statistic	Heterogeneity P value	MR-Egger intercept	Intercept P value	MR-PRESSO global test P value
Osteoporosis	Imaging-based LFC	IVW	17	12.341	0.779	4.086e-05	0.749	0.833
		MR-Egger	16	12.235	0.728			
	cALT	IVW	59	87.963	0.009	-7.830	0.390	0.011
		MR-Egger	58	86.839	0.008			
	Biopsy-confirmed NAFLD	IVW	25	41.436	0.021	-9.855	0.370	0.045
		MR-Egger	24	40.043	0.021			

cALT, chronically elevated serum alanine aminotransferase; IVW, inverse variance weighted; MR-Egger, MR-Egger regression; NAFLD, non-alcoholic fatty liver disease.

Using 60 independent SNPs associated with Calt, the two-sample IVW results suggested weak evidence for a causal role of Calt in the risk of OP (OR: 1.001, 95% CI: 1.000-1.002, P = 0.079). Similar directional associations were found with the weighted median, weighted mode and MR Egger methods (OR: 1.002, 95% CI: 1.000-1.003, P = 0.044; OR: 1.002, 95% CI: 1.000-1.003, P = 0.035; OR: 1.002, 95% CI: 1.000-1.005, P = 0.104; **Table 2**, **Figures 2B**, **3A**). Heterogeneity was detected in sensitivity analyses as suggested by Cochran’s Q statistic (Q = 87.963, P = 0.009; **Supplementary Figure 4B**). MR‐PRESSO analysis identified one outlier (rs56094641), and correction of this outlier made the causal estimates become more significant (one outlier removed, OR: 1.001, 95% CI: 1.000-1.002, P = 0.004). “Leave one-out” sensitivity analysis indicated no SNP drove the results (**Supplementary Figure 5B**). The MR-Egger regression indicated no evidence of horizontal pleiotropy (intercept = -7.830e-05, P = 0.390; **Table 3**).

IVW MR analysis for the biopsy‐confirmed NAFLD, including 26 independent SNPs, indicated a causal effect of biopsy‐confirmed NAFLD on the risk of OP (OR: 1.001, 95% CI: 1.000-1.002, P = 0.031), again with consistent results for analyses from the MR-Egger, weighted median and weighted mode methods (**Table 2**, **Figures 2C**, **3A**). Heterogeneity was detected in sensitivity analyses as suggested by Cochran’s Q statistic (Q = 41.436, P = 0.021; **Supplementary Figure 4C**). MR‐PRESSO identified one outlier (rs56094641), and the causal association persisted after excluding this outlier (one outlier removed, OR: 1.001, 95% CI:1.000-1.002, P = 0.004). “Leave one-out” sensitivity analysis indicated no single SNP dominated the causal estimate in the IVW (**Supplementary Figure 5C**). The MR-Egger regression indicated no evidence of horizontal pleiotropy (intercept = -9.855e-05, P = 0.370; **Table 3**).

We also investigated whether severe NAFLD phenotypes, including NASH and liver fibrosis and cirrhosis, lead to the development of OP. However, only one significant SNP (rs3747207) as the genetic IV for NASH or liver fibrosis and cirrhosis was retained after a series of rigorous screenings, which limited the IVW analysis [NASH (OR: 1.001, 95% CI: 1.000-1.002, P = 0.026), liver fibrosis and cirrhosis (OR: 1.002, 95% CI: 1.000-1.003, P = 0.026)].

### Association between genetically predicted NAFLD and OP after correction of confounding risk factors

We searched the human genotype-phenotype association database Phenoscanner V2 (http://www.phenoscanner.medschl.cam.ac.uk/) for associated phenotypes of NAFLD-associated SNPs at the genome-wide significance level to determine whether these SNPs were associated with known risk factors of OP (25).

Several SNPs affecting bone metabolism were observed, including BMI, T2D, smoking, and hypothyroidism. We repeated the analyses after excluding genes that are associated with the mentioned-above risk factors. IVW analysis for imaging-based LFC, with the remaining 14 SNPs, still showed a statistically significant causal association with the risk of OP (OR: 1.003, 95% CI: 1.000-1.005, P = 0.004). Consistently, IVW analysis for Calt and biopsy-confirmed NAFLD, with the remaining 50 and 18 SNPs, respectively, also indicated a significant causal association with the risk of OP (OR: 1.001, 95% CI: 1.000-1.003, P = 0.020; OR: 1.001, 95% CI: 1.000-1.002, P = 0.008). Similar associations were observed when the MR‐Egger, weighted median and weighted mode methods were applied (**Table 2**, **Figure 3B**).

## Discussion

To our knowledge, this is the first study to evaluate the causal relationship between genetically predicted NAFLD and the risk of OP. This study explored the largest database, GWAS, and other relevant databases to investigate the causal associations between three NAFLD-related traits, including imaging-based liver fat, Calt and biopsy-confirmed NAFLD, and the risk of OP. Our findings suggest a causal relationship between NAFLD and the risk of OP. With our efforts to attain the MR assumptions, this study supports that the presence of NAFLD may causally increase the risk of OP.

NAFLD is usually considered as the hepatic manifestation of metabolic syndrome, since it is bidirectionally associated with metabolic comorbidities including obesity, T2D and dyslipidemia (26). NAFLD is also identified as a risk factor for cardiovascular disease, such as hypertension, coronary heart disease, cardiomyopathy and cardiac arrhythmias (27). In NAFLD, an increased accumulation of dysfunctional visceral and ectopic fat, and an activation of inflammatory response, together with the subsequent release of fat-derived toxic metabolites, triggers a series of local and systemic pathophysiological changes that ultimately leads to the development of both hepatic and peripheral metabolic dysfunction (28). Recently, a global multi-society has decided to replace the term NAFLD with “metabolic dysfunction-associated steatotic liver disease” (MASLD), which is defined as the presence of hepatic steatosis along with at least one of five cardiometabolic risk factors that align with the components of metabolic syndrome (29). Scientists further evaluated how the new definition of MASLD impacted disease epidemiology in the US, and found that while the prevalence of disease was similar compared with previous definitions, 90% of the US population could be diagnosed with metabolic dysfunction according to the consensus criteria (30).

Conflicting evidence regarding the association between NAFLD and OP has been obtained thus far. In previous observational studies, it has been reported no association between bone mineral density and NAFLD (14, 15). Inconsistently, recent clinical studies have revealed that NAFLD is significantly associated with an increased risk of OP and osteoporotic fractures (7, 8). The risk of OP or osteoporotic fractures was increased by 33% in patients with NAFLD compared with those without NAFLD (7). However, existing evidence is limited to observational studies, which were all based on case-control or cross-sectional designs, leaving it uncertain whether NAFLD are prospectively associated with an increased risk of OP. In addition, NAFLD and OP share some common risk factors, which may act as the key confounders in determining their independent associations (31), suggesting that they may be linked beyond a simple coincidence. In this study, using genetic variants as instruments, we identified a significant causal linkage between NAFLD and OP, which supports that the presence of NAFLD causally increases the risk of OP. Further repeated MR analyses after excluding genes with significant effects on OP revealed that the causal relationship between NAFLD and OP still preserved after removing the confounding variables, supporting a direct causal effect of NAFLD on OP. In our analyses, there were 2 SNPs (rs58542926 and rs738408) showing more significant SNP effect on exposures, and exclusion of them influenced the significance of the causal effect. Both rs58542926 and rs738408 are well-acknowledged NAFLD-associated SNPs. Scientists have reported that TM6SF2 rs58542926 mutation is one of the important genetic factors leading to NAFLD (32), and it is also associated with advanced hepatic fibrosis/cirrhosis independent of potential confounding factors such as age, BMI, T2D (33). PNPLA3 rs738408 is another genetic factor having strong association with hepatic steatosis, as well as the severity of NAFLD-associated fibrosis/cirrhosis (34, 35). We consider that both of them are important NAFLD-related SNPs and could be used as a proxy of NAFLD, which should be retained in the analyses. This study proved that NAFLD is an active contributor in the pathogenesis of OP. The results suggested an average 0.3% increased risk of OP per SD (5%) higher liver fat by imaging, and that cALT and biopsy-confirmed NAFLD casually increased the risk of OP by 0.1% compared with patients without NAFLD. Although the magnitude of the estimated causal effect is small, we still cannot ignore the impact of NAFLD on OP in clinical practice, especially in patients with NAFLD and concomitant OP and higher fracture risk. Considering that the more severe phenotypes of NAFLD, such as NASH and cirrhosis, may be more strongly associated with OP, large-scale GWAS datasets and additional potentially related genetic variants for severe NAFLD genotypes are required for further validation.

The mechanisms underlying the association between NAFLD and OP have not been well established. We propose that the mechanisms underlying this causal association are as follows: Firstly, in a chronic inflammatory state of liver, the production of pro-inflammatory cytokines, such as IL-6, IL-1 and TNF-α, not only enhance osteoclast genesis and function (36), but also indirectly increase osteoclast activity by promoting the production of nuclear factor kappa-B ligand which is a key player in the pathogenesis of OP (37, 38), thus promoting the process of bone resorption. In this regard, NAFLD, especially NASH characterized as a state of chronic hepatic and systemic inflammation, could adversely affect bone metabolism. Secondly, insulin-like growth factor 1 (IGF-1) is a hepatocyte-derived growth hormone which has anabolic effects on bone growth, through inhibiting osteoblast apoptosis and enhancing osteoblast genesis by stabilizing the Wnt/β-linked protein pathway (39). IGF-1 can also activate mammalian target of rapamycin (Mtor)-induced osteoblast differentiation, migration and chemotaxis, and thus it plays a role in the recruitment of mesenchymal stem cells during bone remodeling (40). Decreased hepatic synthesis function and portal shunt in NAFLD could reduce IGF-1 levels (41), which further decreases osteoblast activity and leads to a bone resorption ratio greater than bone synthesis, thus promoting the development of OP. Thirdly, osteopontin, known as a bone bridging protein which regulates the migration and adhesion of osteoclasts to the bone matrix thus facilitating bone resorption (42), has been shown to be involved in the pathogenesis of NAFLD (43). Previous studies showed that knockout of osteopontin protected mice on a high-fat diet from obesity-induced hepatic steatosis (44), while anti-osteopontin antibodies attenuated NASH and hepatic fibrosis in NASH mouse models (45). Therefore, osteopontin may serve as a mediator in the causal linkage between NAFLD and OP. In addition, altered insulin metabolism in NAFLD may be another important factor that causes both osteoblast and osteoclast malfunctions (46). The concomitant hyperinsulinemia and hyperglycemia in NAFLD could induce osteoblast apoptosis, deteriorate osteoblast proliferation and activity, but enhance osteoclast-mediated bone resorption, leading to uncoupled bone remodeling and slowdown in bone turnover (46, 47). This condition could ultimately lead to inadequate healing of microcracks, poor bone quality and increased fracture risk. On the whole, as a hepatic manifestation of metabolic syndrome, NAFLD may affect OP in several ways, including insulin resistance, chronic inflammatory states, and the release of multiple pro-inflammatory cytokines and bone-affecting molecules. Further experimental studies are needed to clarify the mechanisms underlying the relationships between NAFLD and OP.

Based on the pathophysiological considerations mentioned above, it seems rational that the more severe phenotypes of NAFLD are more strongly associated with OP, mainly due to the chronic, low-grade inflammatory state of NASH and liver fibrosis. However, limited current evidence cannot clarify whether the advanced phenotypes of NAFLD are more closely associated with increased risk of OP than simple liver steatosis, which requires further researches.

Our findings suggest that therapeutics targeting NAFLD might work for OP in clinical practice. Some of the current and emerging medical options for NAFLD have exhibited possible anti-osteoporotic properties, while others have been identified associated with increased risk of fractures and should be avoided in patients with NAFLD and concomitant OP. In view of the causal association between NAFLD and OP, a medication targeting both diseases would be a great advancement. Pioglitazone, recommended as an off-label treatment for NASH, has been reported to be associated with an increased risk of osteoporotic fractures (48). Therefore, this medicine should be avoided in patients with NAFLD and concomitant OP, especially those with higher fracture risk. In contrast, vitamin E, also proposed as an off-label treatment for NASH, has been suggested as a bone-protecting agent via its anti-inflammatory properties, anti-oxidants and promotion of growth factors (49, 50), which should be preferred. Additionally, glucagon-like peptide-1 receptor agonists (GLP-1RAs), which is another class of antidiabetic drug currently under evaluation for NAFLD, has been shown bone-protective effects, probably through the activation of GLP-1R/MAPK signaling pathway, GLP-1R/PI3K/AKT signaling pathway and Wnt/β-catenin pathway. Clinical evidence indicates that GLP-1RA contributes to an improvement of liver histology in NASH patients (51, 52). Meanwhile, it was also indicated increased bone mineral density (53) and reduced fracture risk (54) after receiving GLP-1RAs. Therefore, GLP-1RAs seem to be an appealing therapeutic option for patients with NAFLD and concomitant OP.

This study has several strengths and limitations. The major strength is the MR design which can minimize confounding and reverse causality to a large extent. We extensively mined the largest-scale database, GWAS, to investigate the causal association between exposure and outcome. We used gene‐exposure data of three different NAFLD‐related traits in combination with the use of four different MR methods, contributing to the robustness and validity of our findings. Our results remained overall consistent across several sensitivity analyses. In addition, we confined our analysis to the population of European descent, which effectively reduce the bias caused by the population structure bias. However, the study population of consistent ancestry may limit the generalizability of our findings to other populations. Even though we used several sensitivity analyses to eliminate outlier variants and improve the robustness of the results, horizontal pleiotropy cannot be totally excluded, which means selected genetic IVs influence the risk of outcome not via the exposure but other alternative pathways. In the present study, we detected limited evidence on pleiotropy from MR-Egger intercept test for all traits. Further MR-PRESSO analysis observed few outliers and the association remained consistent or became stronger after removal of outlying SNPs. Another limitation is that the original GWAS was not distinct between different histological stages, therefore, we cannot clarify whether the advanced phenotypes of NAFLD, such as NASH or cirrhosis, are more closely associated with OP. Given that there have only been a few GWAS of NAFLD based on liver biopsy, large-scale GWAS datasets and additional potentially related genetic variants are required for further validation.

In summary, this two‐sample MR study provides evidence to support that NAFLD is a causal risk factor for OP. The presence of NAFLD deserves a thoughtful OP risk assessment in clinical practice, to detect individuals who might benefit from lifestyle and therapeutic interventions aimed at prevention and management of OP and osteoporotic fractures.

## Data availability statement

The datasets presented in this study can be found in online repositories. The names of the repository/repositories and accession number(s) can be found in the article/**Supplementary Material**.

## Ethics statement

Ethical approval was not required for the study involving humans in accordance with the local legislation and institutional requirements. Written informed consent to participate in this study was not required from the participants or the participants’ legal guardians/next of kin in accordance with the national legislation and the institutional requirements.

## Author contributions

YZ: Conceptualization, Data curation, Formal Analysis, Investigation, Methodology, Writing – original draft. YN: Writing – review & editing, Formal Analysis. ZW: Writing – review & editing. GP: Writing – review & editing. QW: Writing – review & editing, Conceptualization, Supervision.

## References

[1] Golob AL Laya MB. Osteoporosis: screening, prevention, and management. Med Clinics North America (2015) 99(3):587–606. doi: 10.1016/j.mcna.2015.01.010 25841602

[2] Xiao PL Cui AY Hsu CJ Peng R Jiang N Xu XH. Global, regional prevalence, and risk factors of osteoporosis according to the World Health Organization diagnostic criteria: A systematic review and meta-analysis. Osteoporosis Int (2022) 33(10):2137–53. doi: 10.1007/s00198-022-06454-3 35687123

[3] Wright NC Looker AC Saag KG Curtis JR Delzell ES Randall S. The recent prevalence of osteoporosis and low bone mass in the United States based on bone mineral density at the femoral neck or lumbar spine. J Bone mineral Res (2014) 29(11):2520–6. doi: 10.1002/jbmr.2269 PMC4757905 24771492

[4] Lewiecki EM Ortendahl JD Vanderpuye-Orgle J Grauer A Arellano J Lemay J. Healthcare policy changes in osteoporosis can improve outcomes and reduce costs in the United States. JBMR plus (2019) 3(9):e10192. doi: 10.1002/jbm4.10192 31667450 PMC6808223

[5] LeBoff MS Greenspan SL Insogna KL Lewiecki EM Saag KG Singer AJ. The clinician's guide to prevention and treatment of osteoporosis. Osteoporosis Int (2022) 33(10):2049–102. doi: 10.1007/s00198-021-05900-y PMC9546973 35478046

[6] Ensrud KE Crandall CJ. Osteoporosis. Ann Intern Med (2017) 167(3):Itc17–itc32. doi: 10.7326/aitc201708010 28761958

[7] Pan B Cai J Zhao P Liu J Fu S Jing G. Relationship between prevalence and risk of osteoporosis or osteoporotic fracture with non-alcoholic fatty liver disease: A systematic review and meta-analysis. Osteoporosis Int (2022) 33(11):2275–86. doi: 10.1007/s00198-022-06459-y 35764892

[8] Su YH Chien KL Yang SH Chia WT Chen JH Chen YC. Nonalcoholic fatty liver disease is associated with decreased bone mineral density in adults: A systematic review and meta-analysis. J Bone mineral Res (2023) 38(8):1092–103. doi: 10.1002/jbmr.4862 37254266

[9] Perumpail BJ Khan MA Yoo ER Cholankeril G Kim D Ahmed A. Clinical epidemiology and disease burden of nonalcoholic fatty liver disease. World J Gastroenterol (2017) 23(47):8263–76. doi: 10.3748/wjg.v23.i47.8263 PMC5743497 29307986

[10] Brunt EM Wong VW Nobili V Day CP Sookoian S Maher JJ. Nonalcoholic fatty liver disease. Nat Rev Dis Primers (2015) 1:15080. doi: 10.1038/nrdp.2015.80 27188459

[11] Younossi Z Tacke F Arrese M Chander Sharma B Mostafa I Bugianesi E. Global perspectives on nonalcoholic fatty liver disease and nonalcoholic steatohepatitis. Hepatology (2019) 69(6):2672–82. doi: 10.1002/hep.30251 30179269

[12] Cho Y Chang Y Ryu S Wild SH Byrne CD. Nonalcoholic fatty liver disease without overlapping metabolic-associated fatty liver disease and the risk of incident type 2 diabetes. Liver Int (2023) 43(11):2445–54. doi: 10.1111/liv.15661 37387519

[13] Targher G Byrne CD Lonardo A Zoppini G Barbui C. Non-alcoholic fatty liver disease and risk of incident cardiovascular disease: A meta-analysis. J Hepatol (2016) 65(3):589–600. doi: 10.1016/j.jhep.2016.05.013 27212244

[14] Ciardullo S Muraca E Zerbini F Manzoni G Perseghin G. Nafld and liver fibrosis are not associated with reduced femoral bone mineral density in the general us population. J Clin Endocrinol Metab (2021) 106(8):e2856–e65. doi: 10.1210/clinem/dgab262 33878156

[15] Upala S Jaruvongvanich V Wijarnpreecha K Sanguankeo A. Nonalcoholic fatty liver disease and osteoporosis: A systematic review and meta-analysis. J Bone mineral Metab (2017) 35(6):685–93. doi: 10.1007/s00774-016-0807-2 27928661

[16] Emdin CA Khera AV Kathiresan S. Mendelian randomization. JAMA (2017) 318(19):1925–6. doi: 10.1001/jama.2017.17219 29164242

[17] Liu Y Basty N Whitcher B Bell JD Sorokin EP van Bruggen N. Genetic architecture of 11 organ traits derived from abdominal Mri using deep learning. eLife (2021) 10:e65554. doi: 10.7554/eLife.65554 34128465 PMC8205492

[18] Vujkovic M Ramdas S Lorenz KM Guo X Darlay R Cordell HJ. A multiancestry genome-wide association study of unexplained chronic alt elevation as a proxy for nonalcoholic fatty liver disease with histological and radiological validation. Nat Genet (2022) 54(6):761–71. doi: 10.1038/s41588-022-01078-z PMC10024253 35654975

[19] Pierce BL Ahsan H Vanderweele TJ. Power and instrument strength requirements for Mendelian randomization studies using multiple genetic variants. Int J Epidemiol (2011) 40(3):740–52. doi: 10.1093/ije/dyq151 PMC3147064 20813862

[20] Hemani G Zheng J Elsworth B Wade KH Haberland V Baird D. The Mr-base platform supports systematic causal inference across the human phenome. eLife (2018) 7:e34408. doi: 10.7554/eLife.34408 29846171 PMC5976434

[21] Qi G Chatterjee N. Mendelian randomization analysis using mixture models for robust and efficient estimation of causal effects. Nat Commun (2019) 10(1):1941. doi: 10.1038/s41467-019-09432-2 31028273 PMC6486646

[22] Burgess S Bowden J Fall T Ingelsson E Thompson SG. Sensitivity analyses for robust causal inference from Mendelian randomization analyses with multiple genetic variants. Epidemiol (Cambridge Mass) (2017) 28(1):30–42. doi: 10.1097/ede.0000000000000559 PMC5133381 27749700

[23] Verbanck M Chen CY Neale B Do R. Detection of widespread horizontal pleiotropy in causal relationships inferred from Mendelian randomization between complex traits and diseases. Nat Genet (2018) 50(5):693–8. doi: 10.1038/s41588-018-0099-7 PMC6083837 29686387

[24] Yavorska OO Burgess S. Mendelianrandomization: an R package for performing Mendelian randomization analyses using summarized data. Int J Epidemiol (2017) 46(6):1734–9. doi: 10.1093/ije/dyx034 PMC5510723 28398548

[25] Kamat MA Blackshaw JA Young R Surendran P Burgess S Danesh J. Phenoscanner V2: an expanded tool for searching human genotype-phenotype associations. Bioinf (Oxford England) (2019) 35(22):4851–3. doi: 10.1093/bioinformatics/btz469 PMC6853652 31233103

[26] Cariou B Byrne CD Loomba R Sanyal AJ. Nonalcoholic fatty liver disease as a metabolic disease in humans: A literature review. Diabetes Obes Metab (2021) 23(5):1069–83. doi: 10.1111/dom.14322 PMC8248154 33464677

[27] Targher G Byrne CD Tilg H. Nafld and increased risk of cardiovascular disease: clinical associations, pathophysiological mechanisms and pharmacological implications. Gut (2020) 69(9):1691–705. doi: 10.1136/gutjnl-2020-320622 32321858

[28] Yki-Järvinen H. Non-alcoholic fatty liver disease as a cause and a consequence of metabolic syndrome. Lancet Diabetes Endocrinol (2014) 2(11):901–10. doi: 10.1016/s2213-8587(14)70032-4 24731669

[29] Rinella ME Lazarus JV Ratziu V Francque SM Sanyal AJ Kanwal F. A multisociety delphi consensus statement on new fatty liver disease nomenclature. Ann Hepatol (2024) 29(1):101133. doi: 10.1016/j.aohep.2023.101133 37364816

[30] Ciardullo S Carbone M Invernizzi P Perseghin G. Exploring the landscape of steatotic liver disease in the general us population. Liver Int (2023) 43(11):2425–33. doi: 10.1111/liv.15695 37592856

[31] Vachliotis ID Anastasilakis AD Goulas A Goulis DG Polyzos SA. Nonalcoholic fatty liver disease and osteoporosis: A potential association with therapeutic implications. Diabetes Obes Metab (2022) 24(9):1702–20. doi: 10.1111/dom.14774 35589613

[32] Xue WY Zhang L Liu CM Gao Y Li SJ Huai ZY. Research progress on the relationship between Tm6sf2 Rs58542926 polymorphism and non-alcoholic fatty liver disease. Expert Rev Gastroenterol Hepatol (2022) 16(2):97–107. doi: 10.1080/17474124.2022.2032661 35057689

[33] Liu Y-L Reeves HL Burt AD Tiniakos D McPherson S Leathart JBS. Tm6sf2 Rs58542926 influences hepatic fibrosis progression in patients with non-alcoholic fatty liver disease. Nat Commun (2014) 5(1):4309. doi: 10.1038/ncomms5309 24978903 PMC4279183

[34] Najafi M Rafiei A Ghaemi A Hosseini V. Association between Rs738408, Rs738409 and Rs139051polymorphisms in Pnpla3 gene and non-alcoholic fatty liver disease. Gene Rep (2022) 26:101472. doi: 10.1016/j.genrep.2021.101472

[35] Anstee QM Daly AK Day CP. Genetic modifiers of non-alcoholic fatty liver disease progression. Biochim Biophys Acta (2011) 1812(11):1557–66. doi: 10.1016/j.bbadis.2011.07.017 21840395

[36] Yang YJ Kim DJ. An overview of the molecular mechanisms contributing to musculoskeletal disorders in chronic liver disease: osteoporosis, sarcopenia, and osteoporotic sarcopenia. Int J Mol Sci (2021) 22(5):2604. doi: 10.3390/ijms22052604 33807573 PMC7961345

[37] Zhong L Yuan J Huang L Li S Deng L. Rankl is involved in runx2-triggered hepatic infiltration of macrophages in mice with Nafld induced by a high-fat diet. BioMed Res Int (2020) 2020:6953421. doi: 10.1155/2020/6953421 32596356 PMC7273465

[38] Anastasilakis AD Toulis KA Polyzos SA Terpos E. Rankl inhibition for the management of patients with benign metabolic bone disorders. Expert Opin investigational Drugs (2009) 18(8):1085–102. doi: 10.1517/13543780903048929 19558335

[39] Locatelli V Bianchi VE. Effect of Gh/Igf-1 on bone metabolism and osteoporsosis. Int J Endocrinol (2014) 2014:235060. doi: 10.1155/2014/235060 25147565 PMC4132406

[40] Xian L Wu X Pang L Lou M Rosen CJ Qiu T. Matrix Igf-1 maintains bone mass by activation of mtor in mesenchymal stem cells. Nat Med (2012) 18(7):1095–101. doi: 10.1038/nm.2793 PMC3438316 22729283

[41] Yang L Yang CQ. Liver cirrhosis and secondary osteoporosis. Zhonghua gan zang bing za zhi = Zhonghua ganzangbing zazhi = Chin J Hepatol (2021) 29(3):204–8. doi: 10.3760/cma.j.cn501113-20210208-00078 PMC12814200 33902185

[42] Yoshitake H Rittling SR Denhardt DT Noda M. Osteopontin-deficient mice are resistant to ovariectomy-induced bone resorption. Proc Natl Acad Sci United States America (1999) 96(14):8156–60. doi: 10.1073/pnas.96.14.8156 PMC22204 10393964

[43] Tang M Jiang Y Jia H Patpur BK Yang B Li J. Osteopontin acts as a negative regulator of autophagy accelerating lipid accumulation during the development of nonalcoholic fatty liver disease. Artif cells nanomedicine Biotechnol (2020) 48(1):159–68. doi: 10.1080/21691401.2019.1699822 31852298

[44] Kiefer FW Neschen S Pfau B Legerer B Neuhofer A Kahle M. Osteopontin deficiency protects against obesity-induced hepatic steatosis and attenuates glucose production in mice. Diabetologia (2011) 54(8):2132–42. doi: 10.1007/s00125-011-2170-0 PMC3131508 21562757

[45] Honda M Kimura C Uede T Kon S. Neutralizing antibody against osteopontin attenuates non-alcoholic steatohepatitis in mice. J Cell communication Signaling (2020) 14(2):223–32. doi: 10.1007/s12079-020-00554-7 PMC7272532 32062834

[46] Wongdee K Charoenphandhu N. Update on type 2 diabetes-related osteoporosis. World J Diabetes (2015) 6(5):673–8. doi: 10.4239/wjd.v6.i5.673 PMC4458496 26069716

[47] Huang S Kaw M Harris MT Ebraheim N McInerney MF Najjar SM. Decreased osteoclastogenesis and high bone mass in mice with impaired insulin clearance due to liver-specific inactivation to Ceacam1. Bone (2010) 46(4):1138–45. doi: 10.1016/j.bone.2009.12.020 PMC2862391 20044046

[48] Viscoli CM Inzucchi SE Young LH Insogna KL Conwit R Furie KL. Pioglitazone and risk for bone fracture: safety data from a randomized clinical trial. J Clin Endocrinol Metab (2017) 102(3):914–22. doi: 10.1210/jc.2016-3237 PMC5460686 27935736

[49] Wong SK Mohamad NV Ibrahim N Chin KY Shuid AN Ima-Nirwana S. The molecular mechanism of vitamin E as a bone-protecting agent: A review on current evidence. Int J Mol Sci (2019) 20(6):1453. doi: 10.3390/ijms20061453 30909398 PMC6471965

[50] Shen CL Yang S Tomison MD Romero AW Felton CK Mo H. Tocotrienol supplementation suppressed bone resorption and oxidative stress in postmenopausal osteopenic women: A 12-week randomized double-blinded placebo-controlled trial. Osteoporosis Int (2018) 29(4):881–91. doi: 10.1007/s00198-017-4356-x 29330573

[51] Mantovani A Petracca G Beatrice G Csermely A Lonardo A Targher G. Glucagon-like peptide-1 receptor agonists for treatment of nonalcoholic fatty liver disease and nonalcoholic steatohepatitis: an updated meta-analysis of randomized controlled trials. Metabolites (2021) 11(2):73. doi: 10.3390/metabo11020073 33513761 PMC7911747

[52] Armstrong MJ Gaunt P Aithal GP Barton D Hull D Parker R. Liraglutide safety and efficacy in patients with non-alcoholic steatohepatitis (Lean): A multicentre, double-blind, randomised, placebo-controlled phase 2 study. Lancet (London England) (2016) 387(10019):679–90. doi: 10.1016/s0140-6736(15)00803-x 26608256

[53] Xie B Chen S Xu Y Han W Hu R Chen M. The impact of glucagon-like peptide 1 receptor agonists on bone metabolism and its possible mechanisms in osteoporosis treatment. Front Pharmacol (2021) 12:697442. doi: 10.3389/fphar.2021.697442 34220521 PMC8243369

[54] Kong QX Ruan Q Fan C Liu BL Reng LP Xu W. Evaluation of the risk of fracture in type 2 diabetes mellitus patients with incretins: an updated meta-analysis. Endokrynologia Polska (2021) 72(4):319–28. doi: 10.5603/EP.a2021.0031 34010433

