# Supplementary material for: Causal effects of non-alcoholic fatty liver disease on osteoporosis: a Mendelian randomization study
Source: Front Endocrinol (Lausanne). 2023 Dec 12;14:1283739. doi: 10.3389/fendo.2023.1283739 (PMC10749958; doi:10.3389/fendo.2023.1283739)

**Supplementary Material 5.** Leave-one-out analysis for NAFLD on risk of OP. A, Imaging-based LFC on OP; B, cALT on OP; C, biopsy-confirmed NAFLD on OP. cALT, chronically elevated serum alanine aminotransferase; LFC, liver fat content; MR, Mendelian randomization; NAFLD, non-alcoholic fatty liver disease; OP, osteoporosis.


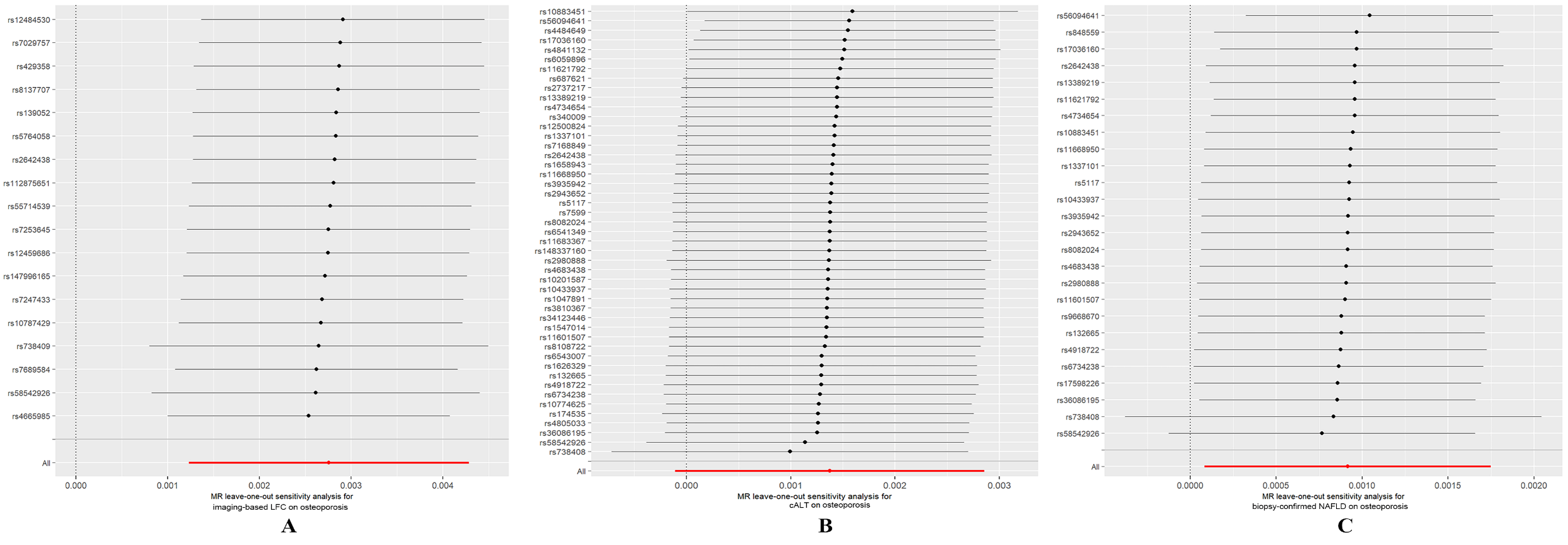

Supplement: Supplementary file 1 [file DataSheet_1.zip › Supplementary Material 5.DOCX]
